# Visceral abdominal fat measured by computer tomography as a prognostic factor for gynecological malignancies?

**DOI:** 10.18632/oncotarget.24667

**Published:** 2018-03-27

**Authors:** Johanna Nattenmüller, Joachim Rom, Tom Buckner, Jalal Arvin, Benedikt Bau, Christof Sohn, Hans-Ulrich Kauczor, Sarah Schott

**Affiliations:** ^1^ Department of Diagnostic and Interventional Radiology, University Hospital, Heidelberg, Germany; ^2^ Department of Obstetrics and Gynecology, University Hospital, Heidelberg, Germany; ^3^ Department of Medical Biometry and Informatics, University Heidelberg, Heidelberg, Germany; ^4^ NCT Heidelberg, DKTK Heidelberg, Heidelberg, Germany; ^5^ Department of Gynecology and Obstetrics, Regional Hospital Nordjylland, Hjørring, Denmark

**Keywords:** body composition, BMI, obesity, gynecological malignancies, computed tomography

## Abstract

**Introduction:**

Obesity is associated with increased incidence of ovarian (OC), cervical (CC) and endometrium cancer (EC). However, the impact of body composition (BC) on overall survival (OS), especially of visceral adipose tissue (VAT) is not yet understood.

**Methods:**

In 189 women with gynecological malignancies (31 OC, 104 CC, 54 EC, mean age 62.9y; mean BMI 26.8 kg/m^2^; median follow-up 30.7months) with routine staging CT-scans at baseline (mean interval: 4.3 months), densitometric quantification of total (TAT), visceral, and subcutaneous-fat-area (SAT), inter-muscular-fat-area (IMFA), and skeletal-muscle-index (SMI) was performed to analyze the impact of BC on survival.

**Results:**

With a mean follow-up of 30.7 months 48 patients had died. We observed no significant differences regarding BMI, the adipose- and muscle-distribution between surviving and deceased women. Univariate analyses revealed no significant BC-parameter with impact on OS, which was confirmed by different multivariate models. A subgroup analysis of OC, CC and EC showed only a protective impact of SMI on survival in the subgroup of CC.

**Conclusions:**

Despite the increased incidence of gynecological malignancies in obese, we found no significant impact of BC including VAT on patient survival. Further studies with larger cohorts are needed to quantify BC and its metabolomic impact regarding treatment and prognosis.

## INTRODUCTION

Overweight and obesity represent a major global health challenge in the 21st century [1]. Half of the population of high-income countries by now is overweight or obese [2]. Worldwide, the incidence has nearly doubled to 12% in adults in 2015 [1]. Obesity is a complex chronic disease with impaired quality of life, high morbidity and mortality, and is an important factor for overall global mortality [1, 3]. In 2015, 7.1% of deaths of any cause were related to high BMI with rising death rates. Overweight and obesity have some of the fastest rising incidences among health risks [1]. Obesity is especially associated with an increased mortality from cardiovascular disease and diabetes, but also from various cancers [1, 2, 4–11]. Yet, the underlying pathophysiology and mechanism to most diseases is not fully understood. Therefore, it is important to develop new therapeutic and prophylactic strategies against obesity to limit health care complications as well as public expenses [1, 12].

Obesity is a risk factor for intra- as well as postoperative complications and remains a considerable, relative contraindication to complex surgical procedures especially in oncological situations [13, 14]. Furthermore, adiposity triggers several comorbidities and limits the use and success of chemotherapeutic and immunological drug regimens [15–17]. The baseline level of inflammation varies in obese and can limit new immunotherapeutic strategies not only due to different responses, but also due to elevated side effects [18].

Obesity is associated with 9% of cancers in female patients [19]. The risk of developing endometrial cancer (EC) has been described as proportional to the second power of the body mass index (BMI) [20]. A meta-analysis including 19 prospective studies showed a strong association between a 5 kg/m^2^ increase in BMI and EC [21]. Every 5 BMI units increase the EC-risk by 50%, and even within the normal BMI range an elevated risk is apparent [22]. Obesity is also associated with an increased risk for all-cause mortality in EC, with the risk being highest for class III obesity (BMI ≥ 40) [23]. Similarly, the incidence of ovarian cancer (OC) and mortality of OC and cervix cancer (CC) is increased in obese [9, 24].

So far, BMI, waist circumference or waist-to-hip ratio at the time of diagnosis have been correlated with prognosis and outcome in gynecological malignancies [25, 26]. But BMI is not able to differentiate between an elevation of fat or muscle tissue. Recently, it has been shown that beyond BMI local fat deposits, especially visceral abdominal fat tissue (VAT), are associated with an increased risk of colon carcinoma [10, 27]. Also, visceral obesity is associated with poor outcome in colorectal cancer patients [28, 29]. Additionally, it is known that tissue composition changes the metabolic, hormonal and digestive properties of a body and might impact drug treatment and cancer metabolome [30].

This study was designed to evaluate the prognostic significance of high VAT in patients with gynecological malignancies. According to our knowledge, this is the first study that examines the exact body composition in these patients.

## RESULTS

In total 189 women were included in this study. Detailed characteristics of the whole study population, including cancer entities and stages, are provided in Table 1. In brief, the average participant was aged 62.9 years, overweighed with an average BMI of 26.8, had given birth (64%) and had no exposure to a hormontherapy in the past (44.4%). Most women underwent chemotherapy (78.3%) as well as radiotherapy (78.8%). Most participants suffered from a cervical cancer (55.0%) followed by endometrium (28.6%) and ovarian cancer (16.4%). Endometrium cancer patients had the highest BMI with (28.4) followed by ovarian cancer patients (26.3), the lowest BMI was found among the cervical cancer patients (26.1). The Rx was classified either if an inoperable situs of an ovarian cancer was detected or if a definite radiochemotherapy was performed.

**Table 1:** Subject characteristics, tumor side and FIGO classification of patients, n=189. Brackets indicate %

General Study Population	
**Total number of patients**	189 (100.0)
Age, year (mean ± SD)	62.88±13.53
BMI (kg/m^2^)	26.81±6.31
**Nulliparity**	
Yes	28 (14.8)
No	121 (64.0)
Unknown	40 (21.2)
**Hormontherapy in anamnesis**	
Yes	25 (13.2)
No	84 (44.4)
Unknown	80 (42.3)
**Chemotherapy**	
Yes	148 (78.3)
No	41 (21.7)
**Radiotherapy**	
Yes	149 (78.8)
No	40 (21.2)
**Radiochemotherapy**	
Yes	100 (52.9)
No	89 (47.1)

Endometrium	54 (28.6)
BMI (kg/m^2^) Mean± SD	28.37±7.49
**FIGO stadium**	
FIGO <IIIC	41 (21.7)
FIGO ≥IIIC	12 (6.3)
unknown	1 (0.5)
**Nulliparity**	
Yes	10 (18.5)
No	35 (64.8)
Unknown	9 (16.7)
**Hormontherapy in anamnesis**	
Yes	7 (13.0)
No	22 (40.7)
Unknown	25 (46.3)
**Chemotherapy**	
Yes	25 (46.3)
No	29 (53.7)
**Radiotherapy**	
Yes	48 (88.9)
No	6 (11.1)
**Radiochemotherapy**	
Yes	11 (20.4)
No	43 (79.6)

Ovarian cancer	31 (16.4)
BMI (kg/m^2^) Mean± SD	26.34±4.40
**FIGO stadium**	
FIGO <IIIC	14 (7.4)
FIGO ≥IIIC	15 (7.9)
unknown	2 (1.1)
**Nulliparity**	
Yes	1 (3.2)
No	20 (64.5)
Unknown	10 (32.3)
**Hormontherapy in anamnesis**	
Yes	5 (16.1)
No	13 (41.9)
Unknown	13 (41.9)
**Chemotherapy**	
Yes	30 (96.8)
No	1 (3.2)
**Radiotherapy**	
Yes	5 (16.1)
No	26 (83.9)
**Radiochemotherapy**	
Yes	5 (16.1)
No	26 (83.9)

Cervical cancer	104 (55.0)
BMI (kg/m^2^) Mean± SD	26.13±6.04
**FIGO stadium**	
FIGO <III	49 (25.9)
FIGO >III	55 (29.1)
**Nulliparity**	
Yes	17 (16.3)
No	66 (63.5)
Unknown	21 (20.2)
**Hormontherapy in anamnesis**	
Yes	13 (12.5)
No	49 (47.1)
Unknown	42 (40.4)
**Chemotherapy**	
Yes	93 (89.4)
No	11 (10.6)
**Radiotherapy**	
Yes	96 (92.3)
No	8 (7.7)
**Radiochemotherapy**	
Yes	84 (80.8)
No	20 (19.2)

The median follow-up time for the last data analysis was 30.7 months (range 7-72 months). At the time point of last data analysis 141 patients (74.2%) were still alive, 48 patients (25.3%) had died.

In the total patient group 34.8 % of patients had sarcopenia, and 65.2 % of patients did not.

In the cervix cancer patient group 34.2 % of patients had sarcopenia, and 65.9% of patients did not.

In the ovarian cancer patient group 32.0 % of patients had sarcopenia, and 68.0 % of patients did not.

In the endometrial cancer patient group 37.3 % of patients had sarcopenia, and 62.8 % of patients did not.

### Distribution of adipose and muscle tissue

Mean values and their standard deviations of TAT, VAT, SAT and VAT/SAT as well as SMI and IMFA quantified at the L3/4 spinal level, are given in Table 2. The mean TAT L3/4 (cm^2^) was 385.2±27.4 cm^2^. In an age-dependent analysis with respect to the median age of 63 years there was only a slight but insignificant difference noticed.

**Table 2:** Total and age stratified distribution (area in cm^2^) of adipose tissue compartments (TAT, VAT, SAT) and SMI at level of lumbal vertebra L3/4, n=189

Variable	Mean± SD	Age< median	Age>median
**TAT L3/4 (cm^2^)**	385.23±27.4	389.3±27.4	381.3±27.4
**VAT L3/4 (cm^2^)**	117.3± 26.6	122.8± 24.89	111.89±28.6
**SAT L3/4 (cm^2^)**	298.5±82.6	311.13±88.4	288.42±69.5
**SMI L3/4 (cm^2^)**	43.1±24.3	42.96± 27.3	43.2±16.9
**IMFA**	37.6±18.7	±	±

SD= standard deviation, TAT=total adipose tissue, VAT=visceral adipose tissue, SAT=subcutaneous adipose tissue, SMI-skeletal-muscle-index, IMFA= inter-muscular-fat-area.

The mean VAT L3/4 (cm^2^) was 117.3± 26.6 and also the age-dependent analysis did not reveal any significant differences. Same results were detected for the SAT L3/4 (cm^2^) and SMI L3/4 (cm^2^) with a mean of 298.5±82.6 and 43.1±24.3, respectively.

### Comparison of surviving vs. deceased patients

In order to analyze the impact of body composition on survival we subgrouped the study population between women still alive versus those who had died. The Satterthwaite t-test performed in order to analyze differences between surviving and deceased patients (total group of all cancer entities) showed no statistical significant differences between the both groups regarding BMI (27.1 vs. 25.9), TAT (399.5 vs. 341.8), VAT (117.6 vs. 115.7), SAT (302.8 vs. 285.1), VAT/SAT (0.47 vs. 0.56), IMFA (37.6 vs. 36.2) as well as SMI (43.6 vs. 41.3). Details are provided in Table 3.

**Table 3:** T-test of surviving (n=142) vs. deceased patients (n=46)

Variable	Alive mean + SD	Deceased mean+ SD	P values alive/deceased
**BMI**	27.1 (6.6)	25.9 ( 5.5)	0.2267
**Age**	62.7 ( 13.3)	63.0 ( 13.9)	0.8904
**TAT L3/4**	399.5 ( 214.1)	341.8 ( 197.1)	0.1014
**VAT L3/4**	117.6 ( 80.7)	115.7 ( 81.2)	0.8908
**SAT L3/4**	302.8 (213.0)	285.1 ( 298.0)	0.7163
**VAT/SAT**	0.47 (0.5)	0.56 (0.3)	0.1714
**SMI L3/4**	43.6(8.0)	41.3 (12.8)	0.2562
**IMFA**	37.6 (18.7)	36.2 (16.7)	0.6249

SD= standard deviation, TAT=total adipose tissue, VAT=visceral adipose tissue, SAT=subcutaneous adipose tissue, SMI-skeletal-muscle-index, IMFA= inter-muscular-fat-area.

### Survival analysis

**Figure 1:**
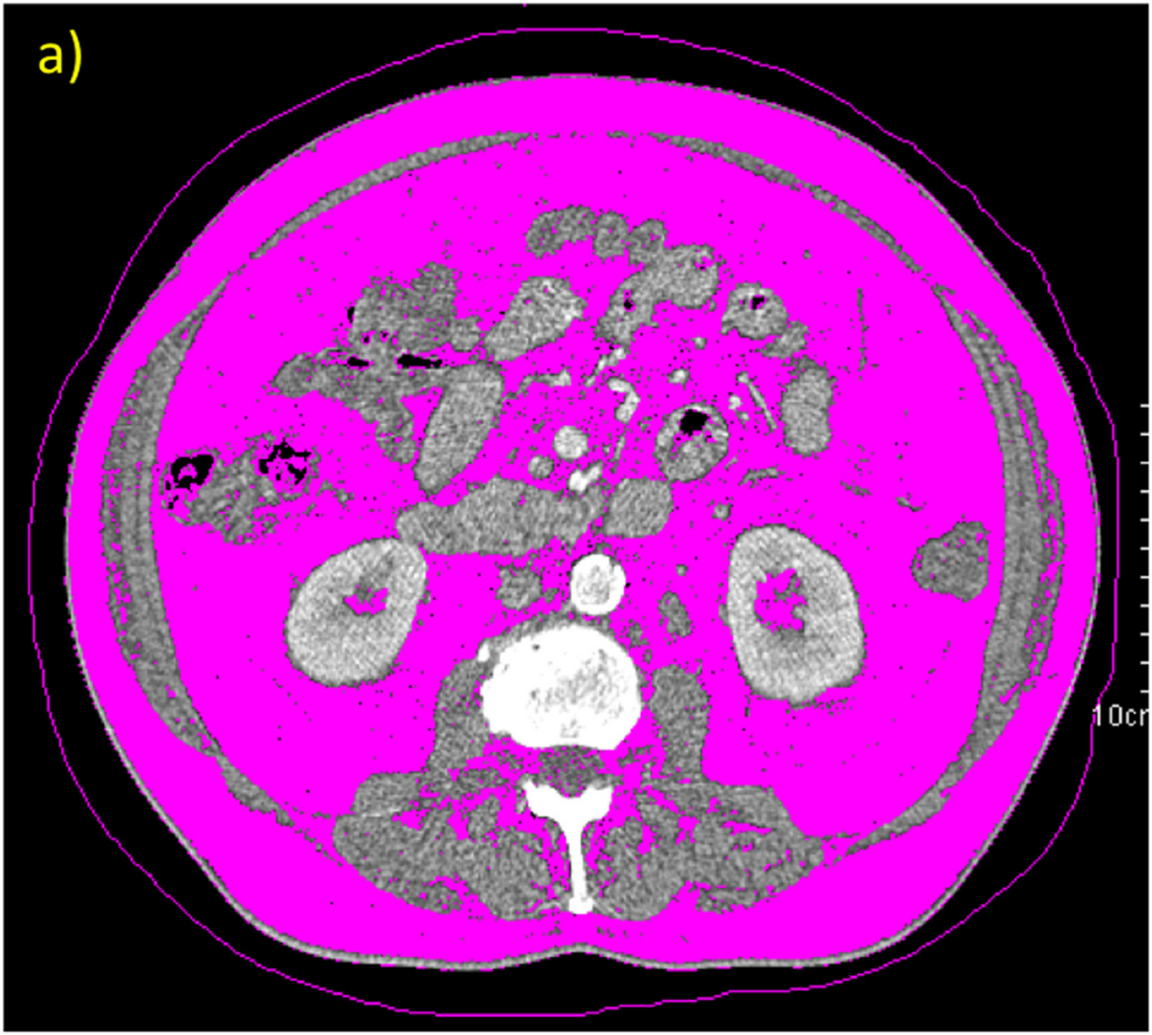

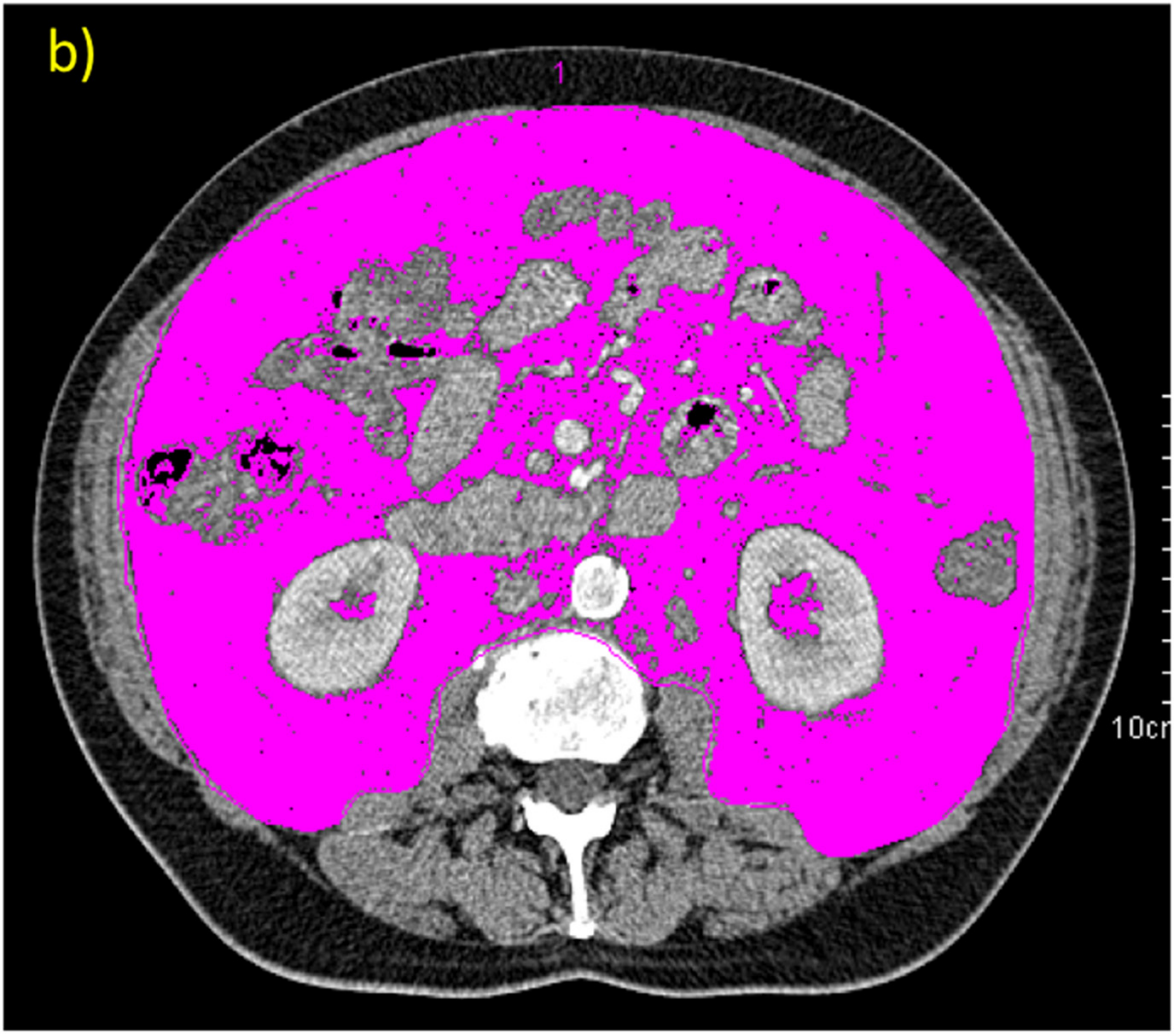

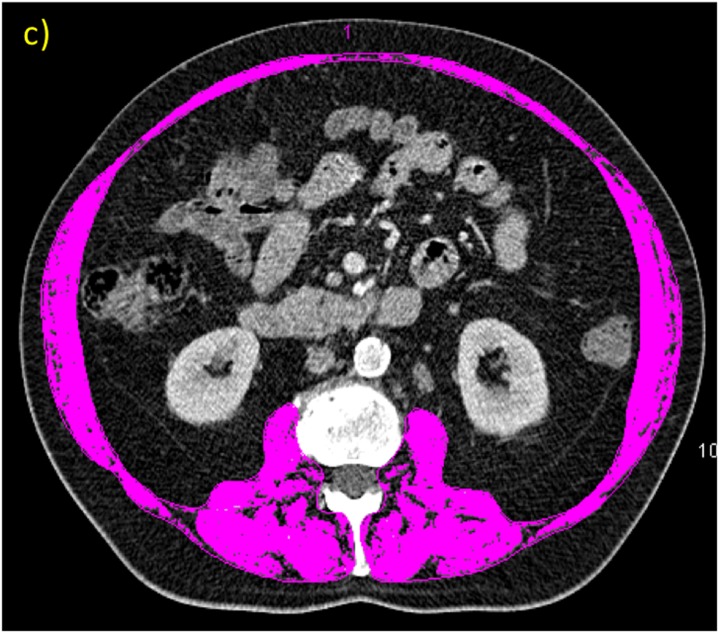

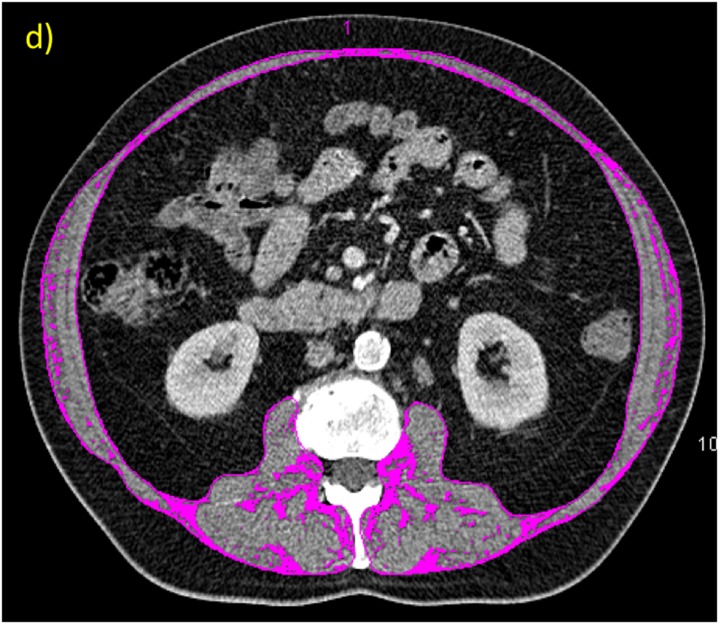
Example of densitometric quantification of adipose tissue area (threshold: -190 to -30 HU) in a CT scan at level L3/4: ROI determining TAT **(a)** and VAT **(b)**. Example of densitometric quantification of muscle area in a CT-scan at level L 3/4 with ROI determining muscle compartments with threshold: -29 to +150 HU **(c)** and IMFA with threshold: -190 to-30HU **(d)**.

The Kaplan-Meier analyses revealed no significant differences between the OC, CC and EC subgroup. Differences on the follow up period were recognized with a median follow-up of 30.7 (range 8-77 months). CC was the lowest among the subgroups (Figure 1).

### Survival and body composition

The univariate Cox regressions were performed to analyze the prognostic impact of the specific body composition on survival. BMI (p=0.3774), TAT (p= 0.1912), VAT (p=0.9164), SAT (p=0.7513), VAT/SAT (p= 0.1167), as well as IMFA (p=0.9366) showed no significant impact on OS in the total study population (see Table 4). SMI has a trend to impact survival with a p-value of p=0.0717, but yet not significant.

**Table 4:** Univariate survival analysis of all study participants with Cox regression, n=189

Variable	Probability Chi-square	Hazard Ratio	Parameter Estimate	95% Hazard Ratio Confidence Limits
**BMI**	0.3774	0.977	-0.02290	0.929/1.028
**Age**	0.8104	1.003	0.00263	0.981/1.024
**TAT L3/4**	0.1912	0.999	-0.00104	0.997/1.001
**VAT L3/4**	0.9164	1.000	0.0001990	0.996/1.004
**SAT L3/4**	0.7513	1.000	-0.000241	0.998/1.001
**VAT/SAT**	0.1167	1.396	0.33384	0.920/2.119
**SMI L3/4**	0.0717	0.975	-0.02560	0.948/1.002
**IMFA**	0.9366	0.999	-0.000670	0.983/1.016
**Subgroup cervix**				
**SMI L3/4**	0.0226	0.959	-0.04158	0.926/0.994

SD= standard deviation, TAT=total adipose tissue, VAT=visceral adipose tissue, SAT=subcutaneous adipose tissue, SMI-skeletal-muscle-index, IMFA= inter-muscular-fat-area.

Also, multivariate analysis performed with various models (formed empirically as well as by forward selection) revealed no significant impact of these parameters on patient’s survival (Supplementary Table 1).

### Subgroup analysis of cancer type

Only in the CC subgroup a high amount of muscle tissue (SMI) was associated with a better survival (p=0.0226, HR=0.959) (Table 4). The univariate Cox regressions of BMI, TFA, VFA, SFA, VFA/SFA, IMFA as well as SMI showed no significant impact on overall survival in the subgroup of OC, EC and CC (excepting SMI), respectively (Supplementary Table 2).

## DISCUSSION

This retrospective study investigated the impact of body composition beyond BMI on the overall survival in women with OC, CC and EC. To this end we conducted an exact quantification of total abdominal adipose tissue including visceral and subcutaneous adipose tissue as well as muscle tissue with an assessment of the skeletal-muscle index (SMI) including intramuscular fat content. Firstly, we investigated the total and age stratified distribution of adipose tissue and found a female specific distribution: the SAT was higher compared to the VAT. This is known from other, non-gynecological malignancies as reported in our previous studies [31]. Secondly, we investigated differences regarding body composition between the groups of surviving versus deceased patients. Here we didn’t find any significant differences in adipose tissue compartments, muscle tissue or BMI. Thirdly, we conducted univariate and multivariate analyses to find possible parameters with impact on overall survival. However, no such significant parameters were found regarding adipose or BMI. SMI showed a general positive trend in all cancers. This means that high amounts of muscle tissue were protective regarding risk of death. Others have shown a significant impact of SMI on survival e.g. in a cohort of lung cancer [32]. The subgroup analysis of cervical cancer goes along with the results from the former mentioned lung cancer study by Nattenmüller et al. as a significant protective impact by SMI was also found. In the subgroups of EC and OC no significant impact of body composition on survival was found. Especially for OC this can be impacted by short follow up period in this subgroup as well as the sample size.

It is known that adiposity as well as gains in body circumference measures are risk factors for cancer occurrence. For example, Aune et al. described a risk increase for EC associated with a ten centimeter increase of waist and hip circumferences as well as waist/hip ratio. Of all gynecologic tumors, EC is most strongly associated with obesity [8, 33]. The large cohort study by Bhaskaran describes relative risks caused by adiposity for CC, OC and EC ranging from 1.09 to 1.62 [33].

In literature, the influence of BMI on outcome in these three analyzed tumors differs, and highlights the individual character of each tumor. Regarding OC, the influence of BMI on prognosis remains questionable [34, 35]. One explanation may be that BMI is not able to reflect the exact body composition. In patients with OC, often cachexia and/or ascites are seen at diagnosis, which also impact the BMI [34]. Also, ascites might indicate a higher FIGO state and does not take other personal factors such as age and race into account [36, 37]. Nevertheless, a higher BMI, measured years before OC-diagnosis, might be a negative prognostic factor [34, 35].

CC and EC seem to be more directly impacted by BMI in respect to outcome. Morbidly obese CC patients have a significantly worse prognosis regarding both OS and cancer-specific survival (CSS) [38]. Data shown in literature, however, are diverse: Frumovitz et al. describe equivalent mortality risks for obese, overweight and normal weight patients [38], whereas other studies showed significantly decreased OS also in obese and overweight patients and a non-significantly decreased CSS [39].

Although an association exists between high BMI in EC patients and positive prognostic histological features such as type I histology and good differentiation, OS significantly decreases with rising BMI [23, 40]. The high obesity-associated cardiovascular mortality in EC-patients, which is 8.8 times higher compared to the general population, is noteworthy in this context [41]. Regarding CSS, a systematic review by Arem and Irving showed no negative prognostic effect of BMI, but few studies exist [40]. A significant negative correlation of CSS with age and BMI among EC patients was noted by Benedetti Pancini et al., revealing a worse CSS for women with a BMI of more than 30 kg/m^2^ aged 65 and above [42].

Tumor-related causes for increased mortality in obese patients may include more limited options in standard diagnostics as well as in therapeutic tools such as lower radiotherapy effectiveness, limitation of chemotherapeutic doses and obesity-specific surgical complications. Also, a lack of screening in obese populations could play a role [39]. Studies examining body composition have shown that lower muscle mass is associated with surgical complications regardless of BMI [37]. Others found higher surgical complication rates in obese EC patients [43, 44]. This in turn might lead to suboptimal treatment in obese patients. Studies on obese cervical cancer patients with early stage disease showed that these populations are less likely to be operated with a radical hysterectomy compared to normal-weight patients [39, 45]. Lower subcutaneous and muscular fat in stage III-IV OC patients were associated with a significantly longer hospital stay and decreased OS [46], which reflects sarcopenia. Accordingly, in the CC-subgroup analyses we found a protective effect of high SMI on survival, which reflects that patients without sarcopenia fare better.

Complications during and after operation delay the onset of adjuvant therapy, which is described among obese patients and known as independent negative prognostic factor [37, 47]. Reduced effect of chemotherapy and therefore worse survival is observed in obese patients, when the dose is “capped” at a certain body surface in order to avoid allegedly higher toxicity - regardless of the actual body surface [23, 35, 38, 39]. Common comorbidities of obese patients might also lead to reduced tolerability [35].

Radiation therapy in obese patients also poses special challenges [23, 38, 48]. As the distance to the tumor is larger, higher energy is needed in obese patients, which may lead to overdose in peripheral soft tissues and excess doses to organs at risk [23, 38, 48]. Furthermore, laxity of the skin can lead to inaccuracies in day-to-day setup [38].

In contrast to those previous cohort studies we assess obesity as well as cachexia in a more personalized and exact manner by measuring body composition directly, independent of the presence of ascites for example. Studies on body composition in other tumor entities showed an impact on oncologic outcomes [36]. Sarcopenia, diagnosed with CT imaging was significantly associated with inferior survival across tumor types and disease stages [36]. Particularly studies on OC showed differing results for the correlation of BMI and disease risk [49] [4, 50]. This might support our finding that fat tissue is more complex and needs further stratification for example by investigating metabolomic and immunological factors in order to create risk profiles that go beyond just BMI and general tumor entity. It also empathizes that the underlying pathomechanisms are more complex and are not fully understood. We hypothesize that there are other factors defining risk than just BMI-measured obesity. Prospective studies are needed to evaluate the composition of different adipose tissues and their influence on prognosis, also addressing tumor cachexia that goes along with a loss of muscle tissue and triggers metastasized disease. Biomarker studies alongside might find predictive factors for the loss of muscle tissue and changes in fat volumes. Previous investigations have shown that elevated inflammation markers, a sign of negative outcome in oncologic patients, are associated with decreased muscle mass but have no association with BMI [51]. Other studies investigating adipocyte-derived free fatty acids have shown a causative link to cancer cell proliferation and invasive properties, which might impact prognosis [52]. Such studies might lead to clinical recommendations, for example the earlier use of parenteral food to increase caloric intake and prevent the metabolomics switch. This also poses the question whether and to what extent the measured fat tissue should be set in relation to muscle tissue, though we could only detect an impact of muscle mass in CC in our cohort. The BMI cannot be principally regarded as a negative prognostic factor as “overweight” patients might for example have higher levels of protective muscle tissue (“obesity paradox”) [53].

This study is subject to the following limitations: as a retrospective study, missing data could not be analyzed, such as smoking status, health status, physical exercise, nutritional habits or cause of death. The differences in fat distribution by race and age were not taken into account. Underweight patients were not excluded and might have led to bias as they also are at higher risk of death due to therapeutic complications and cachexia. As a single center study a selection bias cannot be excluded. CT-scans of different origin and with different protocols were used, however, as all CT-scans were normalized for slice-thickness and adipose tissue quantification is highly reproducible, this should have no impact on our analysis. Because of subgroups with different cancer entities, significant associations in smaller subgroups may be masked.

In conclusion, obesity is a complex disease. The underlying pathomechanisms in cancer patients need further investigation to understand its complexity with respect to cancer development and progression, therapeutic response and its impact on overall prognosis. CT-quantified fat measurements might be a useful tool to examine associations beyond BMI alone. The combination of liquid biopsy, metabolomics, radiomics and exact body composition might be a future approach for an adequate risk assessment among obese cancer patients. Larger studies addressing cancer subtypes are also needed to enhance clinical relevance of such assessments.

## MATERIALS AND METHODS

### Study population and study protocol

This retrospective study conducted at the Department of Gynecology and Obstetrics, University Hospital Heidelberg was approved by the local ethic committee (S-558/2014). All female patients with a gynecological malignancy referred for a surgical treatment to the University Hospital Heidelberg were identified using the medical documentation system (SAP ECC 6.0 EhP5 SP 14 IS-H 605 SP 25) and assessed for eligibility. The inclusion was suitable if CT-scans were available prior to surgery and follow up data were accessible. This was conferred with an extract of the local tumor documentation system, that was also used for survival inquiries. Clinical data relevant for this study were extracted from the medical records. Subjects were categorized upon their tumor entity.

### Quantification of adipose tissue and muscle tissue via CT

We retrieved abdominal CT-scans from the institutional picture and archiving system (PACS, GE Medical Systems, Buckinghamshire, UK). Area-based quantification of adipose tissue compartments was conducted on one representative image slice at level between vertebral-body L3/4 using a semiautomatic software tool (Syngo Volume tool, Siemens Healthcare, Munich, Berlin, Germany) [54]. By manually defining regions of interest (ROI), the total-adipose-tissue (TAT, whole circumference of abdomen) and the visceral-adipose-tissue (VAT, marking the abdominal wall along the fascial plane) were quantified (volumetric measurement of selected slice, divided by slice thickness) as preformed in previous studies [31]. Measurement thresholds with a lower attenuation limit of -190 HU and an upper attenuation limit of -30 HU were chosen to selectively measure adipose tissue within these ROIs as previously described [31, 55]. By subtracting VAT from TAT subcutaneous adipose tissue (SAT) was calculated. The visceral-to-subcutaneous-fat ratio was calculated as VFA/SFA.

**Figure 2:**
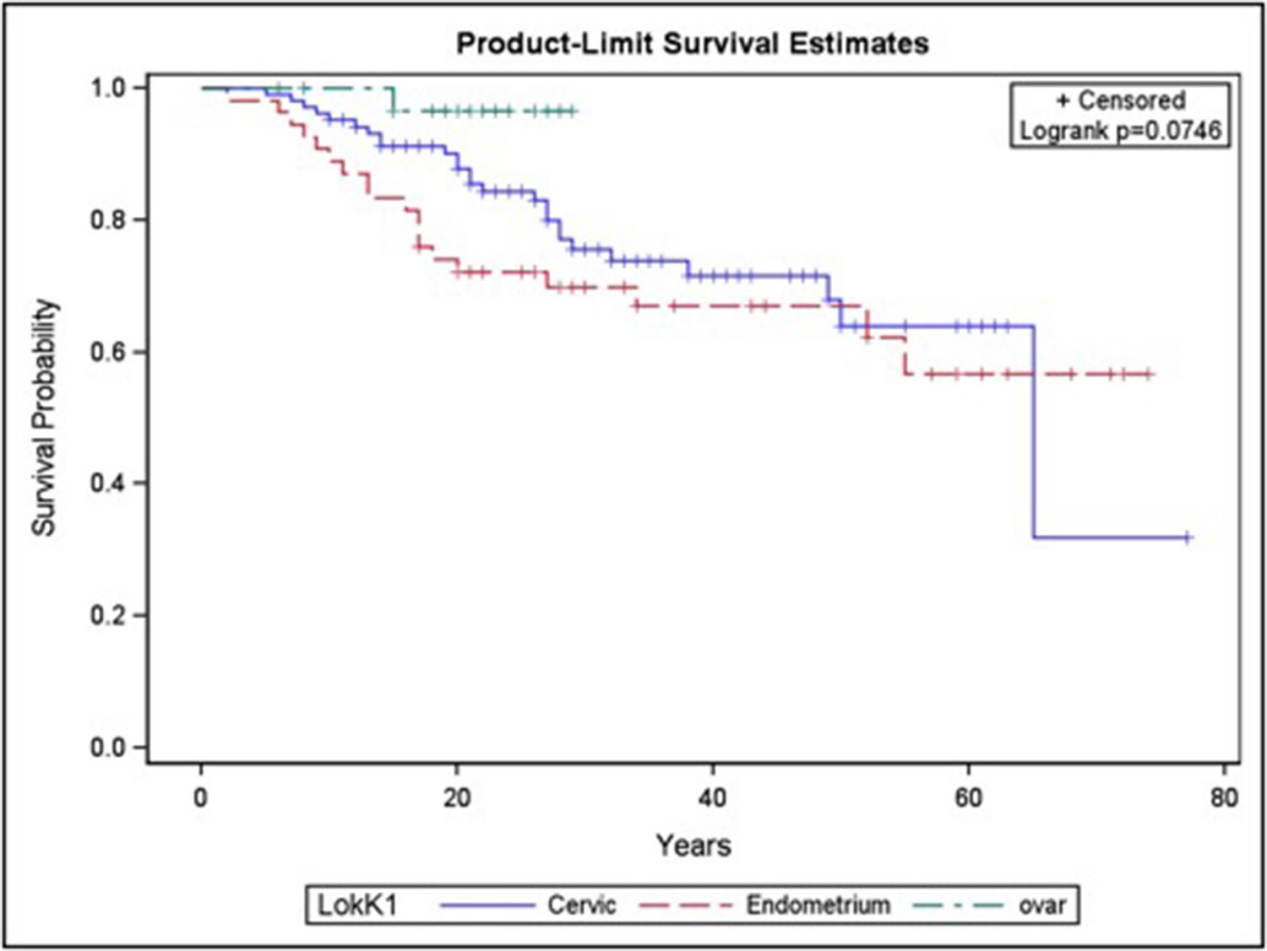
Kaplan-Meier analysis of patients with endometrium (n=54), ovarian (n=31) and cervical cancer (n=104)

On the identical image slice (L3/4) a ROI containing all muscles (M.erector spinae, M.psoas major, M.latissimus dorsi, M.quadratus lumborum, M.transversus abdominis, M.obliquus abdominis externus and internus abdominis and M.rectus abdominis) was manually defined (volumetric quantification of selected slice, divided by slice thickness) [31, 32]. Muscle tissue was selected by limiting the measurements to a lower attenuation limit of -29 HU and an upper attenuation limit of 150 HU (MA_150_, Figure X) [32, 56, 57]. To measure only adipose tissue within muscle compartments (inter-muscular-fat-area, IMFA) a limit of -190 to -30 HU was chosen [32]. Mean muscle density in HU of each ROI was recorded (MD) and skeletal-muscle-index (SMI) was defined as SMI=muscle_150_/(height^2^) with the unit cm^2^/m^2^ [56, 58]. An exemplary image of the CT-based body composition quantification is shown in Figure 2.

According to Martin et al. we defined sarcopenia an SMI <41cm^2^/m^2^ [56].

### Statistical analysis

The statistical analyses were performed using SAS 9.4 (SAS Institute Inc, Cary, NC, US). Continuous data were reported as means with standard deviations and categorical data as absolute and relative frequencies. The Spearman rank correlation coefficient was used to evaluate correlations between ordinal variables, while the t-test and the Wilcoxon U-test was used to test for differences between groups in the case of continuous data or scores. The chi-squared test was performed to evaluate differences between categorical data. Log-rank-test was used to compare survival curves and hazard-ratios were calculated to estimate risks. p < 0.05 (two-sided) was regarded as statistically significant.

## SUPPLEMENTARY MATERIALS TABLES


