# Supplementary material for: Visceral abdominal fat measured by computer tomography as a prognostic factor for gynecological malignancies?
Source: Oncotarget. 2018 Mar 27;9(23):16330–42. doi: 10.18632/oncotarget.24667 (PMC5893243; doi:10.18632/oncotarget.24667)
Supplement: Supplementary file 1 [file oncotarget-09-16330-s001.pdf]

Visceral abdominal fat measured by computer tomography as a prognostic factor for gynecological malignancies?

SUPPLEMENTARY MATERIALS

Supplementary Table 1: Univariate survival analysis with Cox regression in the subgroups of patients with endometrium cancer (a, n=54) and cervical cancer (b, n=104)

a)

Variable	Probability Chi-square	Hazard Ratio	Parameter Estimate	95% Hazard Ratio Confidence Limits
**BMI**	0.6986	0.987	-0.01294	0.925/1.054
**TAT L3/4**	0.2058	0.998	-0.00171	0.996/1.001
**VAT L3/4**	0.8080	0.999	-0.0006421	0.994/1.005
**SAT L3/4**	0.5749	1.001	0.0005518	0.999/1.002
**VAT/SAT**	0.1970	2.296	0.83126	0.649/8.119
**SMI L3/4**	0.4261	0.981	-0.01882	0.937/1.028
**IMFA**	0.4993	1.008	0.00771	0.985/1.031

b)

Variable	Probability Chi-square	Hazard Ratio	Parameter Estimate	95% Hazard Ratio Confidence Limits
**BMI**	0.1665	0.938	-0.06353	0.858/1.027
**TAT L3/4**	0.1931	0.998	-0.00166	0.996/1.001
**VAT L3/4**	0.5715	0.998	-0.00209	0.991/1.005
**SAT L3/4**	0.2510	0.998	-0.00153	0.996/1.001
**VAT/SAT**	0.4238	1.293	0.25672	0.689/2.425
**SMI L3/4**	**0.0226***	**0.959**	**-0.04158**	**0.926/0.994**
**IMFA**	0.1683	0.978	-0.02252	0.947/1.010

SD= standard deviation, TAT=total adipose tissue, VAT=visceral adipose tissue, SAT=subcutaneous adipose tissue, SMI-skeletal-muscle-index, IMFA= inter-muscular-fat-area. *: $p<0.05$.
There was not performed a Cox regression in ovarian cancer patients, as there were no deceased patients in this entity.

Supplementary Table 2: Multivariate survival analysis of all study participants, n=189: model 1 with parameters age, SMI, IMFA and VAT (a) and model 2 with parameters BMI, Age, VAT, SAT, VAT/SAT, IMA and SMI (b)

a)

Variable	Probability Chi-square	Hazard Ratio	Parameter Estimate	95% Hazard Ratio Confidence Limits
**Age**	0.7119	1.005	0.00485	0.979/1.031
**SMI L3/4**	0.5295	0.987	-0.01301	0.94/1.028
**IMFA**	0.7396	0.996	-0.00399	0.973/1.020
**VAT L3/4**	0.8151	1.001	0.0006004	0.996/1.006

b)

Variable	Probability Chi-square	Hazard Ratio	Parameter Estimate	95% Hazard Ratio Confidence Limits
**BMI**	0.3288	0.952	-0.04894	0.863/1.051
**Age**	0.8875	1.002	0.00234	0.970/1.035
**VAT L3/4**	0.8788	1.001	0.0005130	0.994/1.007
**SAT L3/4**	0.4121	1.001	0.0009187	0.999/1.003
**VAT/SAT**	0.4204	1.260	0.23078	0.718/2.208
**SMI L3/4**	0.5098	1.037	0.03613	0.931/1.154
**IMFA**	0.8829	0.998	-0.00221	0.969/1.028

VAT=visceral adipose tissue, SAT=subcutaneous adipose tissue, SMI-skeletal-muscle-index, IMFA= inter-muscular-fat-area. [*]: $p<0.05$.
